# Long-term outcomes of phakic patients with diabetic macular oedema treated with intravitreal fluocinolone acetonide (FAc) implants

**DOI:** 10.1038/eye.2015.98

**Published:** 2015-06-26

**Authors:** Y Yang, C Bailey, F G Holz, N Eter, M Weber, C Baker, S Kiss, U Menchini, J M Ruiz Moreno, P Dugel, A Lotery

**Affiliations:** 1 Royal Wolverhampton Hospitals NHS Trust, Wolverhampton, UK; 2 University Hospitals Bristol NHS Foundation Trust, Bristol, UK; 3 University of Bonn, Bonn, Germany; 4 University of Muenster Medical Center, Muenster, Germany; 5 University Hospital of Nantes, Nantes, France; 6 Paducah Retinal Center, Paducah, KY, USA; 7 Weill Cornell Medical College, New York, NY, USA; 8 Università degli Studi di Firenze, Firenze, Italy; 9 University Castilla La Mancha, Albacete, Spain; 10 Retinal Consultants of Arizona, Phoenix, AZ, USA; 11 University of Southampton, Southampton, UK

## Abstract

**Purpose:**

Diabetic macular oedema (DMO) is a leading cause of blindness in working-age adults. Slow-release, nonbioerodible fluocinolone acetonide (FAc) implants have shown efficacy in the treatment of DMO; however, the National Institute for Health and Care Excellence recommends that FAc should be used in patients with chronic DMO considered insufficiently responsive to other available therapies only if the eye to be treated is pseudophakic. The goal of this analysis was to examine treatment outcomes in phakic patients who received 0.2 *μ*g/day FAc implant.

**Methods:**

This analysis of the phase 3 FAME (Fluocinolone Acetonide in Diabetic Macular Edema) data examines the safety and efficacy of FAc implants in patients who underwent cataract extraction before (cataract before implant (CBI) group) or after (cataract after implant (CAI) group) receiving the implant. The data were further examined by DMO duration.

**Results:**

Best corrected visual acuity (BCVA) after 36 months was comparable in the CAI and CBI groups. Both the percentage of patients gaining ≥3 lines of vision and mean change in BCVA letter score were numerically greater in the CAI group. In addition, most patients who underwent cataract surgery experienced a net gain in BCVA from presurgery baseline as well as from original study baseline.

**Conclusions:**

These data support the use of 0.2 *μ*g/day FAc implants in phakic as well as in pseudophakic patients. These findings will serve as a pilot for design of future studies to evaluate the potential protective effect of FAc implants before cataract surgery in patients with DMO and cataract.

## Introduction

Diabetic macular oedema (DMO) is the most common cause of vision loss in the working-age population, and its chronic nature can necessitate ongoing treatment to maintain vision.^1, 2, 3^ Recent landmark studies have shown that frequent intravitreal injections with inhibitors of vascular endothelial growth factor (VEGF) were more effective than laser photocoagulation for the treatment of centre-involving DMO.^4, 5, 6, 7, 8, 9, 10, 11, 12^ Despite the visual and anatomical improvements seen in many patients treated with anti-VEGF agents, a sizeable proportion of patients still have a poor response, even with frequently repeated injections. In the phase 3 ranibizumab studies for DMO (RISE and RIDE), between 30.4 and 43.2% of patients failed to gain ≥10 letters after 3 years of treatment with 0.3 or 0.5 mg ranibizumab.^4^ In the Diabetic Retinopathy Clinical Research Network (DRCRnet) Protocol I study, at the 3-year visit, 57.6% of patients in the ranibizumab+prompt laser group and 44.2% in the ranibizumab+deferred laser group failed to gain ≥10 letters, with 6% and 3%, respectively, losing ≥15 letters.^5^ In this study, a subanalysis showed that ≈20% of patients could be defined as nonresponsive to ranibizumab therapy, with a consistently <20% reduction in central retinal thickness compared with baseline after repeated injections over a 1-year period.^13^ Thus, not all patients with DMO experience a good and sufficient response with repeated anti-VEGF therapy.

Intravitreal steroids have been evaluated in DMO because of their ability to act on other inflammatory cytokines and pathogenetic mechanisms in addition to those associated with VEGF. Bioerodible DEX implants that release drug for 3 to 6 months were examined in the PLACID and MEAD studies.^14, 15^ The PLACID study showed best corrected visual acuity (BCVA) improvements (proportion of patients gaining ≥10 letters) in patients with diffuse DMO treated with the DEX implant+laser photocoagulation *vs* photocoagulation alone after 9 months but did not meet its primary end point of greater improvements at month 12.^14^ In the MEAD study, the percentage of patients with 15-letter improvement in BCVA from baseline at study end was greater with the DEX implant 0.7 mg (22.2%) than with sham (12.0% *P*=0.018). Rates of cataract-related adverse events (AEs) in phakic eyes were 67.9% *vs* 20.4% in the DEX implant 0.7 mg group *vs* sham group, respectively, and 2 patients (0.6%) in the DEX implant 0.7 mg group required incisional surgery for elevated intraocular pressure.^16^

Slow-release, nonbioerodible implants of fluocinolone acetonide (FAc) were evaluated in patients with DMO who had received previous laser photocoagulation in the FAME (Fluocinolone Acetonide in Diabetic Macular Edema) study; these implants were found to be more efficacious than the standard of care at 3 years after a single intravitreal injection.^17, 18^ Approximately a third (34%) of patients with DMO for >3 years who were treated with 0.2 *μ*g/day FAc implant experienced a ≥15-letter gain compared with 13.4% of patients treated with sham. The results of this study led to worldwide marketing authorizations of the 0.2 *μ*g/day FAc implant for the treatment of chronic DMO that has been insufficiently responsive to available therapies.^19^

Despite the promising prospect for FAc implants to play a major role in the long-term therapy of patients with chronic DMO, there is still a concern over the issue of cataract formation following intravitreal corticosteroid therapy.^18, 20^ This concern was reflected in the recent guidance by the National Institute for Health and Care Excellence (NICE) that FAc should be used in patients with chronic DMO considered insufficiently responsive to other available therapies only if the eye to be treated is already pseudophakic. However, in clinical practice, chronic DMO in phakic eyes with and without cataract is often encountered, and the risk of developing worsening DMO in the early postoperative period following cataract surgery is significantly higher, especially in patients who had previous treatment for DMO.^21, 22, 23, 24, 25, 26, 27^ It is therefore of value to investigate the outcome of FAc in phakic eyes with chronic DMO. In this study, a *post hoc* analysis of FAME study data was undertaken to evaluate the functional and anatomical outcomes at 3 years in patients who underwent cataract surgery during follow-up against those who were already pseudophakic at baseline.

## Materials and methods

The detailed design and methodology of the FAME study have been described previously. Briefly, the study consisted of two phase 3, randomized, double-masked, sham injection-controlled, parallel-group, multicentre trials (FAME A and B; www.clinicaltrials.gov, NCT00344968) performed under a single protocol.^17, 18^ The trials adhered to the guidelines of the Declaration of Helsinki and were approved by each institution's governing institutional review board or ethics committee.

In the FAME study, after patients with DMO gave written informed consent, they were screened for eligibility by having their BCVA and foveal centre point thickness (CPT) measured, as previously described.^17, 18, 28^ Eligible patients had BCVA between 19 and 68 Early Treatment Diabetic Retinopathy Study (ETDRS) letters and foveal thickness ≥250 *μ*m despite ≥1 prior focal/grid macular laser photocoagulation treatment. Detailed inclusion and exclusion criteria were reported previously.^17, 18^ A total of 956 patients were randomized 2 : 2 : 1 to treatment in a single eye with 0.2 *μ*g/day FAc, 0.5 *μ*g/day FAc, or sham injection (control). The full analysis population (*N*=956) consisted of all randomized patients and was used for efficacy determination. The full safety population (*n*=953) consisted of all patients receiving ≥1 study treatment; prespecified subgroups were examined to determine whether certain patient populations had an expanded benefit-to-risk ratio. Subgroup populations presented herein (chronic and nonchronic DMO) consisted of all patients with a duration of DMO≥or< the median duration of all study participants, respectively. The median baseline duration of DMO in the FAME study, as calculated by the prespecified algorithm ((year of randomization)−(year of diagnosis)+1), was 3 years. This was the prespecified algorithm to determine median duration of DMO at baseline; however, the robustness of this finding was tested with a sensitivity analysis exploring the differential treatment effect when the duration of DMO was calculated using the day/month/year for diagnosis and randomization. This yielded a median DMO duration of 1.73 years. Because the differential treatment effect seen with the preplanned algorithm was replicated, with the algorithm providing a more exact determination of median duration of DMO at baseline (*κ*=0.8508), results in the present analysis used the prespecified algorithm, yielding a median DMO of 3 years.

Timing of study visits was presented previously.^17, 18^ Lens status was determined by the assessing physician before randomization. Patients underwent cataract surgery by phacoemulsification technique at the discretion of the masked investigator. No guidance was given regarding the timing of cataract surgery.

In this *post hoc* analysis, only patients treated with 0.2 *μ*g FAc were included, as the number of patients treated with sham injections undergoing cataract surgery was small (*n*=32).

### Statistical analyses

Patients were initially divided into two main subgroups according to whether their study eye underwent cataract extraction before (cataract before implant (CBI) group) or after (cataract after implant (CAI) group) receiving the implant. Each subgroup was then subdivided into two further subgroups according to whether the duration of DMO in the study eye was <3 years (nonchronic) or ≥3 years (chronic).

Comparisons were not tested for significance because of the *post hoc* nature of the analyses. Efficacy and safety after cataract surgery were run as observed case analyses, using only data available at the observed time points without imputing any values for missing patient data. Changes in visual acuity were summarized relative to the last presurgical assessments to characterize the effects of cataract surgery. Other outcomes included foveal thickness changes, fluorescein leakage, and AEs following cataract surgery that were observed during the FAME study follow-up period.

## Results

### Demographics

**Figure 1:**
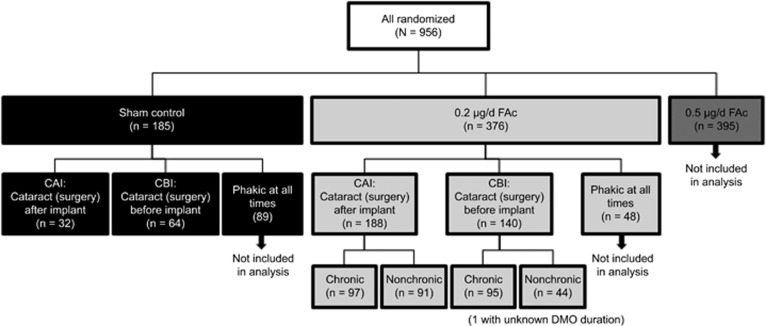
Flow-through study by lens status and duration of DMO. DMO, diabetic macular oedema; FAc, fluocinolone acetonide.

In the FAME trials, 956 patients in the full safety population were randomized to either 0.2 *μ*g/day FAc (*n*=376), 0.5 *μ*g/day FAc (*n*=395), or sham injection (control; *n*=185) in one study eye (Figure 1). The mean age of the FAME trial population was 62.5 years. Among those receiving 0.2 *μ*g/day FAc, 328 patients were pseudophakic; of these, 140 had cataract surgery before receiving the FAc implant (CBI group) and 188 had cataract surgery after receiving the implant (CAI group). The mean age of patients receiving cataract surgery before receiving the implant (CBI group) was 67.7; mean age for patients receiving cataract surgery after receiving the implant (CAI group) was 60.5 years, respectively (*P*<0.0001). The baseline characteristics in terms of BCVA, CPT, duration and type of diabetes, and glycosylated haemoglobin were similar across all four subgroups (Table 1). In the CBI group, median time to development of cataract was 12 months; median time to cataract extraction was 18 months.

### Visual acuity outcomes

When all patients treated with 0.2 *μ*g/day FAc were examined, visual acuity after 36 months was comparable in patients who had cataract surgery after (CAI group) and in those who had cataract surgery before (CBI group) receiving the implant.

**Figure 2:**
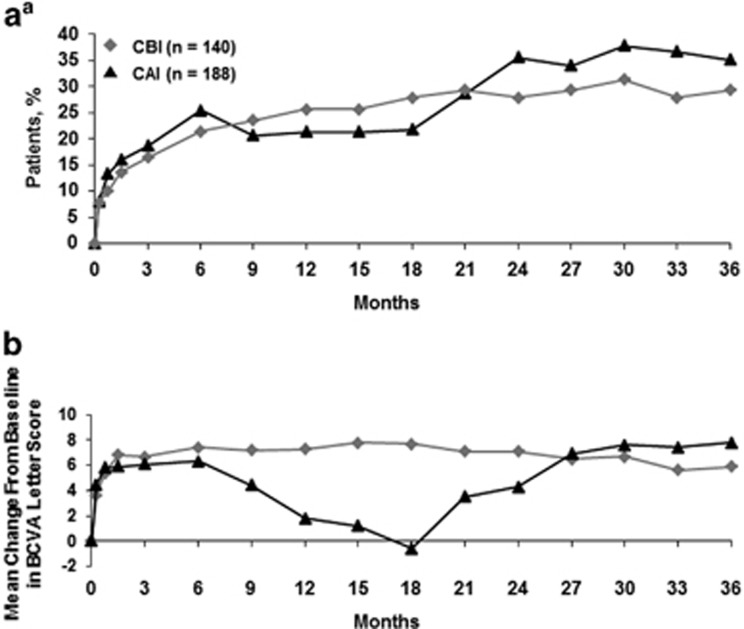
Visual acuity in 0.2 *μ*g/day FAc-treated patients as a function of lens status. (a) Proportion of patients experiencing a ≥15-letter improvement in BCVA and (b) mean change in BCVA letter score. BCVA, best corrected visual acuity; CAI, cataract (surgery) after implant; CBI, cataract (surgery) before implant; FAc, fluocinolone acetonide. ^a^Integrated full analysis population.

Visual outcome (the proportion of patients who experienced a ≥15-letter improvement) at month 36 was slightly higher overall in the CAI group (35.1%) than in the CBI group (29.3% Figure 2a). When the change in BCVA letter score from baseline to month 36 was considered, patients in the CAI group experienced a decline in BCVA between months 6 and 18 as expected because of cataract formation. Patients in the CBI group did not experience this dip and maintained their visual gain from month 3 (Figure 2b). However, at 36 months, the change in BCVA was numerically greater in the CAI group. Of note, most pseudophakic patients enrolled in the FAME trials had undergone cataract surgery >9 months before randomization, a time considered long enough for inflammation because of cataract surgery to resolve. When BCVA outcome was analysed as a function of time in patients whose cataract surgery occurred within 9 months *vs* outside this window, no signal was seen that would support an increased effect in patients whose surgery occurred closer to randomization.

**Figure 3:**
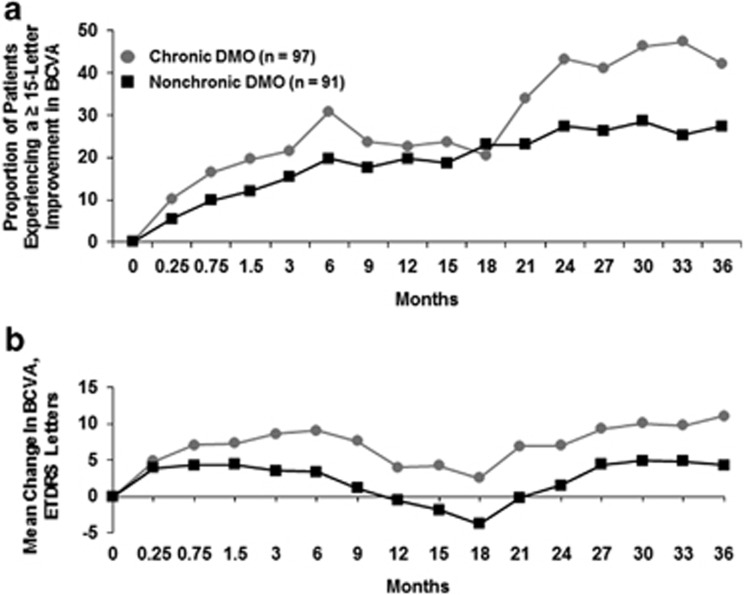
Visual acuity in 0.2 *μg*/day FAc-treated patients who developed cataract during the FAME trials (CAI group). (a) Proportion of patients experiencing a ≥15-letter improvement in BCVA and (b) mean change in BCVA letter score. BCVA, best corrected visual acuity; CAI, cataract (surgery) after implant; CBI, cataract (surgery) before implant; FAc, fluocinolone acetonide; FAME, Fluocinolone Acetonide in Diabetic Macular Edema.

When only patients who had cataract surgery during the study (CAI group) were evaluated, patients with chronic DMO were more likely to gain a ≥15-letter improvement than those with nonchronic DMO (42.3% *vs* 27.5%, respectively; Figure 3a). Improvements in mean BCVA letter score were also greater in chronic *vs* nonchronic patients (11.1 *vs* 4.3 letters, respectively; Figure 3b).

**Figure 4:**
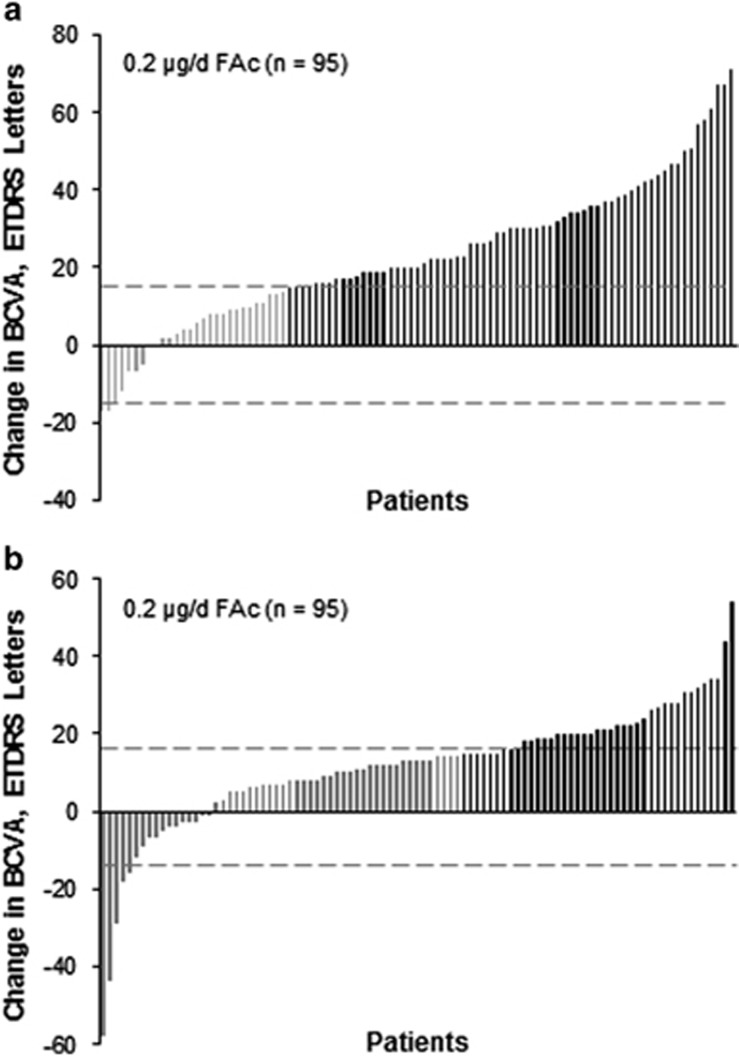
Change in visual acuity following cataract surgery in 0.2 *μ*g/day FAc-treated patients with chronic DMO (a) from the last presurgical BCVA letter score to the last postsurgical BCVA letter score and (b) from original study baseline to the last postsurgical BCVA letter score. BCVA, best corrected visual acuity; DMO, diabetic macular oedema; ETDRS, Early Treatment Diabetic Retinopathy Study; FAc, fluocinolone acetonide. Black lines indicate improvement of ≥15 letters.

The visual gain in patients with chronic DMO in the CAI group can also be illustrated individually using a waterfall plot, as shown in Figure 4. The majority of patients who underwent cataract surgery experienced a net gain in BCVA from presurgery baseline (measured at the last time point directly preceding surgery; Figure 4a).The maximum gain among these patients was +77 letters and the maximum loss was −14 letters. Vision improvement was also noted after cataract surgery relative to original study baseline, as shown in Figure 4b.

### Angiographic and morphological changes

For the full population, the change from baseline in fluorescein leakage at month 36 was similar between CBI and CAI patients (−2.1 and −1.7 disc areas, respectively). Moreover, values were similar regardless of DMO chronicity. Among CBI patients with chronic DMO, the change from baseline was −2.0 disc areas *vs* −2.4 disc areas for CBI patients with nonchronic DMO. Among CAI patients, the change in fluorescein leakage at month 36 slightly favoured chronic *vs* nonchronic patients (−2.1 *vs* −1.3 disc areas). When these anatomical outcomes following cataract surgery in CAI patients treated with 0.2 *μ*g/day FAc were examined specifically, there was an increase in fluorescein leakage at 6 months post procedure (1.21 disc areas); this was reduced to a 0.55–disc area increase at 12 months post procedure from presurgery baseline. In contrast, fluorescein leakage remained relatively constant for patients receiving sham at 6 and 12 months post procedure (2.4 and 2.37 disc areas, respectively).

In addition, in patients treated with 0.2 *μ*g/day FAc who underwent cataract surgery, CPT showed a small increase immediately after surgery that quickly recovered by month 3 and was stable to month 12 in patients with chronic DMO (mean CPT of 287 *μ*m at the last visit before cataract surgery; 365 *μ*m at 1 month post surgery; 297 *μ*m at 3 months post surgery). In patients with nonchronic DMO, the stabilization did not occur until month 9 (mean CPT of 308 *μ*m at the last visit before cataract surgery; 355 *μ*m at 1 month post surgery and returned to presurgery values by month 9 (312 *μ*m). These findings suggest that macular anatomy was not disturbed following cataract surgery, especially in patients with chronic DMO who had received 0.2 *μ*g/day FAc.

### AEs after cataract surgery

AEs were uncommon following cataract surgery. Corneal oedema—which occurred in 7 of 188 patients (3.7%) in the 0.2 *μ*g/day FAc implant group, 8 of 231 (3.5%) in the 0.5 *μ*g/day FAc implant group, and 0 of 33 in the sham control group—was transient and mild. Among patients receiving 0.2 *μ*g/day FAc who underwent laser capsulotomy, 9 of 11 had postcapsulotomy follow-up ranging from 197 to 666 days, with 2 patients undergoing the procedure just before exiting the study. Of the 9 patients with long-term follow-up, no migration of the implant into the anterior chamber was reported. All other events studied—including wound dehiscence, cataract operation complication, eye inflammation, and anterior chamber flare—occurred in two or fewer patients.

## Discussion

The FAc implant was found to be cost effective by NICE in patients with chronic DMO considered insufficiently responsive to other therapies, provided the eye to be treated was already pseudophakic. The reason for the stipulation on the pseudophakic status of the treated eye was because of differences in cost effectiveness between treating phakic and pseudophakic eyes.^29^ Although this guidance has been very helpful for the clinician who is faced with a patient whose DMO has been unresponsive to other therapies, it has also resulted in an unmet need for patients with chronic DMO who are phakic, with or without cataract. In addition, it has raised the question of whether it is actually less clinically beneficial to use FAc in phakic eyes and then perform cataract surgery subsequently if and when the cataract worsens or develops. The FAME study is the largest study to date on the treatment of DMO using the 0.2 *μ*g/day FAc implant. Given the comparable numbers of patients who were phakic and pseudophakic at baseline and the availability of long-term prospective follow-up data, the study provides a useful opportunity to compare long-term outcomes of patients with DMO undergoing cataract surgery after implantation (CAI) with those undergoing cataract surgery before implantation (CBI). Results in patients who had cataract surgery during the FAME trials showed that visual outcomes of phakic eyes treated with 0.2 *μ*g/day FAc were no worse and possibly better than visual outcomes seen in patients with pseudophakic eyes. When postcataract surgery outcomes in patients who had surgery during the study were examined, these findings suggest a possible protective effect of following treatment with 0.2 *μ*g/day FAc on macular function in eyes that have to undergo cataract surgery.

Cataract occurred at an expectedly high rate in phakic patients treated with the FAc implant, as this is a known effect of any intravitreal corticosteroid therapy. However, in contrast to previous reports that have demonstrated an exacerbation of DMO and worsening of vision following cataract extraction in this population, the results from this *post hoc* analysis suggest that visual outcomes in these patients were not negatively affected by cataract surgery. Among patients receiving 0.2 *μ*g/day FAc implants, more phakic patients who underwent cataract surgery during the study experienced numerically higher gains in mean BCVA and a ≥15-letter improvement than those who were pseudophakic at baseline. This differential in visual acuity outcome was even more pronounced in patients with chronic DMO.

When outcomes following cataract surgery were examined, patients treated with 0.2 *μ*g/day FAc implants experienced an increase in BCVA following surgery that continued for 1 year. This is in contrast to reports that patients with more advanced DMO experience poor visual outcomes after cataract surgery.^25, 26, 30, 31, 32^ Overall, these findings suggest that the continuous exposure of low-dose corticosteroid provided by 0.2 *μ*g/day FAc implants may provide a protective effect in the period directly following and/or during cataract surgery as well as favourable long-term visual outcomes.

It has been reported that inflammatory factors are upregulated after cataract extraction, the presence of which could contribute to worsening of DMO and decreased vision.^22, 33^ A low dose of corticosteroid present during and after surgery could ameliorate this influx of inflammatory factors and associated sequelae that in turn may account for some of the differences seen between the 0.2 *μ*g/day FAc group and sham control group. Because some retina specialists may delay cataract surgery in patients with DMO based on the potential for exacerbation, the presence of 0.2 *μ*g/day FAc implant may influence clinical behaviour. The protective effect noted among patients treated with 0.2 *μ*g/day FAc implants who underwent cataract surgery and the vision gains noted among those who became pseudophakic with an implant in the eye may therefore support the implantation of this device in patients with chronic DMO regardless of lens status. Of importance is the observation that in patients treated with the 0.2 *μ*g/day FAc implant who underwent cataract surgery and subsequent capsulotomy, there was no evidence of migration of the implant into the anterior chamber.

One of the limitations of this study was the *post hoc* nature of the analyses. As such, these results were not powered to detect differences between patients receiving sham and those treated with 0.2 *μ*g/day FAc. The observed case analysis of the postcataract surgery mean change in BCVA favoured the 0.2 *μ*g/day FAc-treated patients. However, there was a significant difference in the age of participants in the CAI and CBI groups that could have affected the results and this underscores the need for prospective study on this topic. It is also important to note that the majority of patients in the FAME study were probably already presbyopic at baseline and would not have lost accommodative function as a result of cataract surgery. For younger patients with chronic DMO who still have natural accommodative power and no cataract formation or symptoms, it may be more appropriate to defer 0.2 *μ*g/day FAc intravitreal implant until it is clear that an adequate response with anti-VEGF therapy cannot be achieved.

In conclusion, these data support the use of 0.2 *μ*g/day FAc implants in both pseudophakic and phakic eyes of patients with chronic and nonchronic DMO. Phakic eyes with chronic DMO treated with FAc and requiring subsequent cataract surgery had particularly favourable visual outcomes. These findings will be of value to clinicians in justifying the use of FAc in phakic eyes of patients with DMO that has been unresponsive to other therapies and will serve as a pilot for the design of future studies to evaluate whether there is any protective effect of a FAc implant before cataract surgery in patients with DMO and cataract.


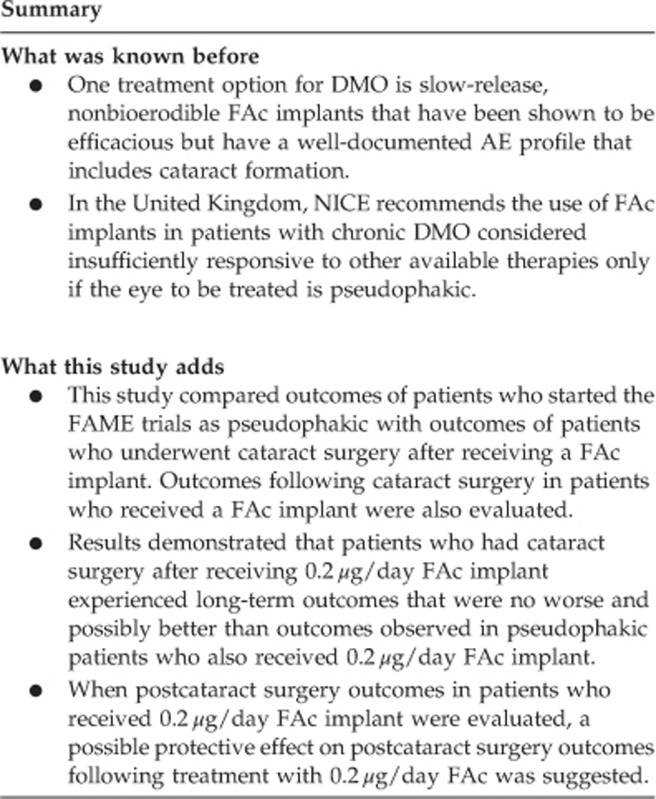


## Figures and Tables

**Table 1:** Baseline characteristics of 0.2 *μ*g/day FAc-treated patients by lens status and duration of DMO

	*Pseudophakic at baseline (CBI group)*		*Phakic→pseudophakic (CAI group)*	
	*Chronic DMO (*n=*95)*	*Nonchronic DMO (*n=*44)*	*Chronic DMO (*n=*97)*	*Nonchronic DMO (*n=*91)*
BCVA, mean (SE), ETDRS letters	52.5 (1.40)	53.3 (2.00)	51.6 (1.33)	54.7 (1.19)
Centre point thickness, mean (SE), *μ*m	448.0 (17.45)	490.0 (22.59)	462.0 (17.11)	463.2 (16.43)
Duration of diabetes, mean (SE), y	19.7 (0.98)	18.2 (1.54)	17.2 (0.93)	15.0 (0.91)
Duration of DMO, mean (SE), y	5.1 (0.31)	1.6 (0.08)	5.2 (0.33)	1.7 (0.05)
				
*Diabetes type,* n *(%)*				
Type 1	8 (8.4)	3 (6.8)	9 (9.3)	4 (4.4)
Type 2	85 (89.5)	41 (93.2)	85 (87.6)	86 (94.5)
Uncertain	2 (2.1)	0	3 (3.1)	1 (1.1)
HbA_1c_, mean (SE), %	7.6 (0.16)	7.8 (0.17)	7.9 (0.17)	7.9 (0.19)

Abbreviations: BCVA, best corrected visual acuity; CAI, cataract (surgery) after implant; CBI, cataract (surgery) before implant; DMO, diabetic macular oedema; ETDRS, Early Treatment Diabetic Retinopathy Study; FAc, fluocinolone acetonide; HbA_1c_, glycosylated haemoglobin; SE, standard error.

## Notes

YY: honoraria, advisory board: Alimera Sciences, Allergan, Alcon, Bayer, Novartis, Thrombogenics; consultancy: Alimera Sciences, Allergan; CB: honoraria: Novartis; commercial funding for clinical trial recruitment via Bristol Eye Hospital: Alimera Sciences; research funding for clinical trials work via Bristol Eye Hospital: Bayer; FGH: research funding, consultancy, grants, honoraria: Alimera Sciences, Allergan, Novartis; NE: research funding, speakers bureau, honoraria, advisory board: Novartis, Bayer, Allergan; speakers bureau, honoraria: Heidelberg Engineering; advisory board: Bausch and Lomb; research funding, advisory board: Alimera Sciences; MW: honoraria, advisory board: Alimera Sciences; CB: advisory board: Alimera Sciences; SK: consultancy, honoraria: Alimera Sciences, Allergan, Genentech/Roche, Regeneron, speakers bureau: Allergan, Genentech/Roche, Regeneron; PD: consultancy, honoraria: Alimera Sciences, Allergan, Alcon; AL: research funding, honoraria, advisory committee: Alimera Sciences; JMRM and UM: nothing to disclose.
